# Supplementary material for: Content-rich biological network constructed by mining PubMed abstracts
Source: BMC Bioinformatics. 2004 Oct 8;5:147. doi: 10.1186/1471-2105-5-147 (PMC528731; doi:10.1186/1471-2105-5-147)
Supplement: Additional File 2 — The original results of the above study (non-essential files are deleted to keep the file size under the limit set by BMC bioinformatics). [file 1471-2105-5-147-S2.bz2 › chilibotAdditionalFile2/dip05/674ID10767348E11080/html/ACVRL1_ENG.html]

 


 **ACVRL1** and **ENG** 
  
Found 524 abstracts in PubMed, retrieved 05.  
 

 What does Google say? 
 PDF only 
| .edu only 

---

**Interactive relationship** (e.g. stimulation, inhibition, etc)

**Neutral relationship**- Thromboxane A 2 synthase TXAS binds to the endoplasmic reticulum membrane and catalyzes both an isomerization of prostaglandin H 2 PGH 2 to form thromboxane A 2 TXA 2 and a fragmentation of PGH 2 to form 12 hydroxy, 10 heptadecatrienoic acid HHT  [ **ENG** ]   [ **ACVRL1** ]  and malondialdehyde MDA.  Ref: 12859980 Arch Biochem Biophys, 2003
- HHT  [ **ENG** ]   [ **ACVRL1** ]  types 1 and 2 are caused by loss of function mutations in  **ENG**  and  **ACVRL1** .  Ref: 12812985 Hum Mol Genet, 2003
- The two known disease types, HHT1  [ **ENG** ]  and HHT2  [ **ACVRL1** ] , are caused by mutations in the endoglin  [ **ENG** ]   **ENG**  and ALK 1  [ **ACVRL1** ]  genes, respectively.  Ref: 12843319 J Med Genet< MedlineTA>J Med Genet, 2003

**Non-interactive relationship** (e.g. studied together, co-existance, homology, etc.)

- The identification of ALK 1  [ **ACVRL1** ]  mutations in subjects with a suspected diagnosis and without clinical signs of HHT  [ **ENG** ]   [ **ACVRL1** ]  argue in favour of a molecular diagnosis.  Ref: 12843319 J Med Genet< MedlineTA>J Med Genet, 2003
- Purpose To describe the angiographic features of hepatic involvement in hereditary hemorrhagic telangiectasia HHT  [ **ENG** ]   [ **ACVRL1** ] , particularly the presence of portovenous shunts.  Ref: 12813620 Cardiovasc Intervent Radiol< MedlineTA>Cardiovasc Intervent Radiol, 2003
- The transforming growth factor beta signaling family is a key player in genetic and multifactorial diseases, including hereditary hemorrhagic telangiectasia HHT  [ **ENG** ]   [ **ACVRL1** ] , cancer, atherosclerosis and immunomodulation.  Ref: 12812985 Hum Mol Genet, 2003
- Hereditary haemorrhagic telangiectasia HHT  [ **ENG** ]   [ **ACVRL1** ]  is a genetic vascular disorder characterised by epistaxis, telangiectases, and visceral manifestations.  Ref: 12843319 J Med Genet< MedlineTA>J Med Genet, 2003
- Substrate specificity of the alpha L arabinofuranosidase from Rhizomucor pusillus HHT  [ **ENG** ]   [ **ACVRL1** ]  1.  Ref: 12829392 Carbohydr Res, 2003
- Methods We reviewed the angiographic findings of seven patients with HHT  [ **ENG** ]   [ **ACVRL1** ] .  Ref: 12813620 Cardiovasc Intervent Radiol< MedlineTA>Cardiovasc Intervent Radiol, 2003
- A higher frequency of pulmonary arteriovenous malformations AVMs has been reported for HHT1  [ **ENG** ]  while HHT2  [ **ACVRL1** ]  is thought to be associated with a lower penetrance and milder disease manifestations.  Ref: 12843319 J Med Genet< MedlineTA>J Med Genet, 2003
- These findings stress the need for an early diagnosis of HHT  [ **ENG** ]   [ **ACVRL1** ]  that can be useful for the early control of associated visceral involvement.  Ref: 12843319 J Med Genet< MedlineTA>J Med Genet, 2003
- The TXAS 2C5 chimera contains heme at nearly a 1 1 molar ratio and catalyzes the formation of TXA 2, MDA, and HHT  [ **ENG** ]   [ **ACVRL1** ]  at a 1 1 1 ratio, although with a reduced catalytic activity compared to wild type TXAS.  Ref: 12859980 Arch Biochem Biophys, 2003
- The alpha L arabinofuranosidase AF from the fungus Rhizomucor pusillus HHT  [ **ENG** ]   [ **ACVRL1** ]  1 released arabinose at appreciable rates from 1 >5 alpha L arabinofuranooligosaccharides, sugar beet arabinan and debranched arabinan.  Ref: 12829392 Carbohydr Res, 2003
- Conclusions Hepatic vascular lesions in HHT  [ **ENG** ]   [ **ACVRL1** ]  are varied, ranging from telangiectasias to large shunts between three vascular channels.  Ref: 12813620 Cardiovasc Intervent Radiol< MedlineTA>Cardiovasc Intervent Radiol, 2003
